# Airborne Bacterial Deposition Onto Surgical Sites Under Operating Room Versus Field Sterility: A Passive Air Sampling Study Across 3 Surgical Environments

**DOI:** 10.1177/15589447261467940

**Published:** 2026-07-29

**Authors:** Erin Hopkins, Joshua Gillis

**Affiliations:** 1 Memorial University of Newfoundland, St. John’s, Canada; 2 Division of Plastic Surgery, NL Health Services, St. John’s, Canada

**Keywords:** airborne contamination, passive air sampling, surgical site infections, field sterility, operating room, minor procedures

## Abstract

**Background:**

Airborne microbial contamination contributes to surgical site infections. While operating rooms (ORs) use specialized ventilation, many minor procedures are now performed outside the main suite using field sterility. This study compared airborne bacterial deposition across 3 clinical settings with differing sterility protocols.

**Methods:**

An observational environmental sampling study was conducted in a minor procedures room, a day surgery suite, and a main OR. Minor procedures and day surgery used identical field-sterility draping; the OR used full sterile draping. On each sampling day, 15 blood agar settle plates were exposed in 3 sequential 1-hour intervals (5 plates/hour) on a tray 1 m above the floor and 1 m from the operative field, following index of microbial air contamination principles. After 24-hour incubation at 37 °C, colony-forming units (CFUs) were counted (n = 180 total; 60 per environment). Mean CFU counts were compared using 1-way analysis of variance (ANOVA) with Tukey post hoc testing.

**Results:**

Overall differences in mean CFU counts across the 3 environments were statistically significant (1-way ANOVA, *F* = 23.10, *P* < .001). The minor procedures room had the highest mean CFU/plate at 3.48 (*SD* = 2.53), followed by the day surgery suite at 2.23 (*SD* = 1.47), and the OR at 1.17 (*SD* = 1.38). Tukey post hoc testing showed significantly higher counts in the minor procedures room compared with the OR (mean difference [MD] = 2.32, *P* < .0001) and day surgery suite (MD = 1.25, *P* = .0013). Day surgery counts were also significantly higher than the OR (MD = 1.07, *P* = .0058).

**Conclusions:**

Airborne microbial deposition differed significantly across environments, lowest in the OR and highest in the minor procedures room. Despite identical field-sterility draping in minor procedures and day surgery, contamination levels varied, suggesting environmental factors beyond draping influence microbial burden and should be considered when evaluating procedural environments.

## Introduction

Airborne contamination is a recognized route of transmission for pathogenic microorganisms in health care environments.^1-4^ Surgical and procedural spaces therefore incorporate environmental controls to reduce the risk of surgical site infections (SSIs).^2^,^5-7^ Surgical site infections represent a major global public health challenge, accounting for approximately 20% of all health care–associated infections and contributing significantly to patient morbidity and mortality.^8^ Evidence has demonstrated a correlation between airborne microbial burden and postoperative infection risk,^9^ prompting ongoing debate about the necessity of operating room sterility for all procedures.^10-13^ As a result, understanding how airborne microbial deposition varies across different clinical environments has become an important area of investigation.

This question is particularly relevant in hand and wrist surgery, where many procedures are increasingly performed outside the main operating room.^10,11,14,15^ Hand and wrist procedures such as carpal tunnel release, trigger finger release, Kirschner-wire fixation, and excision of small skin lesions are commonly performed in minor procedure rooms or day surgery suites rather than in a formal operating room environment. Leblanc et al^14^ reported that more than 70% of Canadian carpal tunnel releases are performed outside the main operating room under field sterility with surgeon-administered local anesthesia, with infection rates of 0.4% for superficial infection and 0% for deep infection. These findings suggest that the safety of minor procedure room surgery may not require the full infrastructure of operating room sterility and highlight that the cost and environmental burden of main operating room sterility may not always be necessary for such procedures.

Operating rooms typically incorporate features such as laminar airflow, high-efficiency ventilation, and restricted access to maintain low airborne microbial counts.^12,13,16^ However, these outpatient procedures are commonly performed under “field sterility,” with standard skin preparation and draping but without the infrastructure of a formal operating theater.^10,11,14,15^ Several studies have reported low infection rates in minor procedures and WALANT (wide-awake local anesthesia no tourniquet) surgeries performed in outpatient or minor surgery settings.^14,15^ These findings have challenged traditional assumptions regarding the necessity of full operating room sterility for all cases, yet the role of airborne microbial deposition in these differing environments remains poorly characterized.

The present study quantified airborne bacterial deposition across 3 surgical environments with differing sterility protocols: a minor procedures room, a day surgery suite, and a main operating room. Using passive air sampling to measure colony-forming units (CFUs), we compared microbial deposition across these settings. This allowed evaluation of whether environments employing identical field-sterility draping demonstrate similar airborne microbial burdens and helped identify settings in which full operating room infrastructure may be unnecessary.

## Methods

This observational environmental sampling study was conducted in March and April 2025 across 3 distinct hospital environments in Eastern Canada: a minor procedures room, a day surgery suite, and a main operating room. Sampling was performed on separate designated days each week to ensure consistency of setting and workflow.

On each sampling day, 15 sterile blood agar plates (Hardy Diagnostics, Santa Maria, California; 5% sheep blood in tryptic soy agar [TSA], 15 × 100 mm) were exposed in 3 sets of 5. Plates were placed on a stainless-steel surgical tray positioned approximately 1 m above the floor and 1 m from the operative field, consistent with the index of microbial air contamination (IMA) method.^17^ Each set of plates was exposed for exactly 1 hour, yielding 3 hours of sampling per environment per day. Plate exposure occurred during active clinical workflow, including ongoing surgical or procedural activity. Sampling was not intentionally performed during terminal room cleaning or prolonged periods of inactivity, although routine room entry, staff movement, and standard between-case workflow were not restricted. When not in use, plates were stored inverted and flat at room temperature. All agar plates were labeled in advance to ensure accurate site and time tracking.

Following exposure, plates were incubated at 37 °C for 24 hours within 5 hours of collection. Colony-forming units were counted manually for each plate, and data were recorded by plate, hourly interval, and environment. In total, 180 plates were analyzed, with 60 plates collected from each environment across 4 sampling days per environment over a 4-week period. No effort was made to alter normal workflow, room entry, or traffic during sampling to reflect real-world contamination conditions.

Patient preparation and draping procedures were standardized within each sterility setting. In the minor procedures room and the day surgery suite, procedures were performed under field sterility using a consistent towel-drape technique (4 sterile green towels) following standard skin preparation. In contrast, the operating room used full operating room sterility, including full sterile draping, restricted access, and laminar airflow, in addition to standard patient preparation. In all environments, surgeons wore standard procedural attire including surgical masks, caps, sterile gloves, and sterile gowns according to institutional practice.

For statistical analysis, CFU counts were recorded for each plate and organized by environment and sampling interval. Descriptive statistics were calculated for each environment, including mean, standard deviation (*SD*), median, mode, and interquartile range. Differences in mean CFU counts across the 3 environments were evaluated using a 1-way analysis of variance (ANOVA). When overall differences were identified, Tukey’s honestly significant difference (HSD) post hoc testing was performed to assess pairwise comparisons between environments. Normality was assessed prior to ANOVA analysis using visual inspection of data distribution and residuals. A formal a priori power calculation was not performed, as this study was exploratory and intended to characterize airborne microbial deposition across clinical environments using repeated passive sampling.

## Results

A total of 180 blood agar plates were exposed across 3 clinical environments during the 4-week sampling period, with 60 plates collected from each environment, as shown in Table 1. Each site was sampled once weekly, with 15 plates collected per sampling day (5 plates per hour over 3 hours). Complete plate-level CFU counts for all sampling days are provided in the supplemental material.

**Table 1.** Total Number of Blood Agar Plates Collected Across 3 Hospital Environments During March to April 2025.

Environment	Sampling days (n)	Plates per day (n)	Total plates (n)
Minor procedures room	4	15	60
Day surgery suite	4	15	60
Operating room	4	15	60
Total	—	—	180

**Figure 1.**
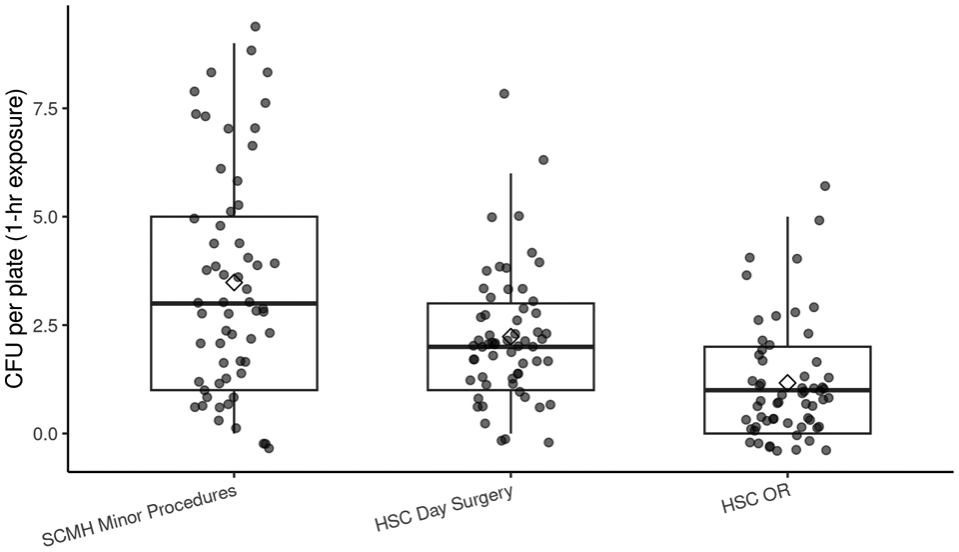
Comparison of airborne bacterial loads (CFU/plate) between St. Clare’s Mercy Hospital (SCMH) minor procedures, Health Sciences Centre (HSC) day surgery, and Health Sciences Centre (HSC) operating room settings in Eastern Canada. Box = interquartile range; horizontal line = median; diamond = mean; points = individual settle plates. CFU indicates colony-forming unit; OR, operating room.

Mean CFU counts differed across environments, as illustrated in Figure 1. The minor procedures room demonstrated the highest average contamination at 3.48 CFU per plate (*SD* = 2.53), followed by the day surgery suite at 2.23 CFU per plate (*SD* = 1.47), while the operating room exhibited the lowest contamination at 1.17 CFU per plate (*SD* = 1.38). The distribution of CFU counts by environment is summarized in Table 2. Overall differences in mean CFU counts across the 3 environments were statistically significant (1-way ANOVA, *F* = 23.10, *P* < .001), as shown in Table 3.

**Table 2.** CFU Counts per Plate by Hospital Environment, Presented as Mean CFU/Plate (*SD*), Range, and Total Number of Plates Analyzed.

Environment	Mean CFU/plate (*SD*)	Range	N plates
Minor procedures room	3.48 (2.53)	0-9	60
Operating room	1.17 (1.38)	0-6	60
Day surgery suite	2.23 (1.47)	0-8	60

Abbreviation: CFU, colony-forming unit.

**Table 3.** One-Way ANOVA Results Comparing CFU Counts Across Environments.

Test	*F* statistic	df (between, within)	*P*-value
One-way ANOVA	23.10	(2, 177)	<.001

Abbreviations: ANOVA, analysis of variance; CFU, colony-forming unit.

**Figure 2.**
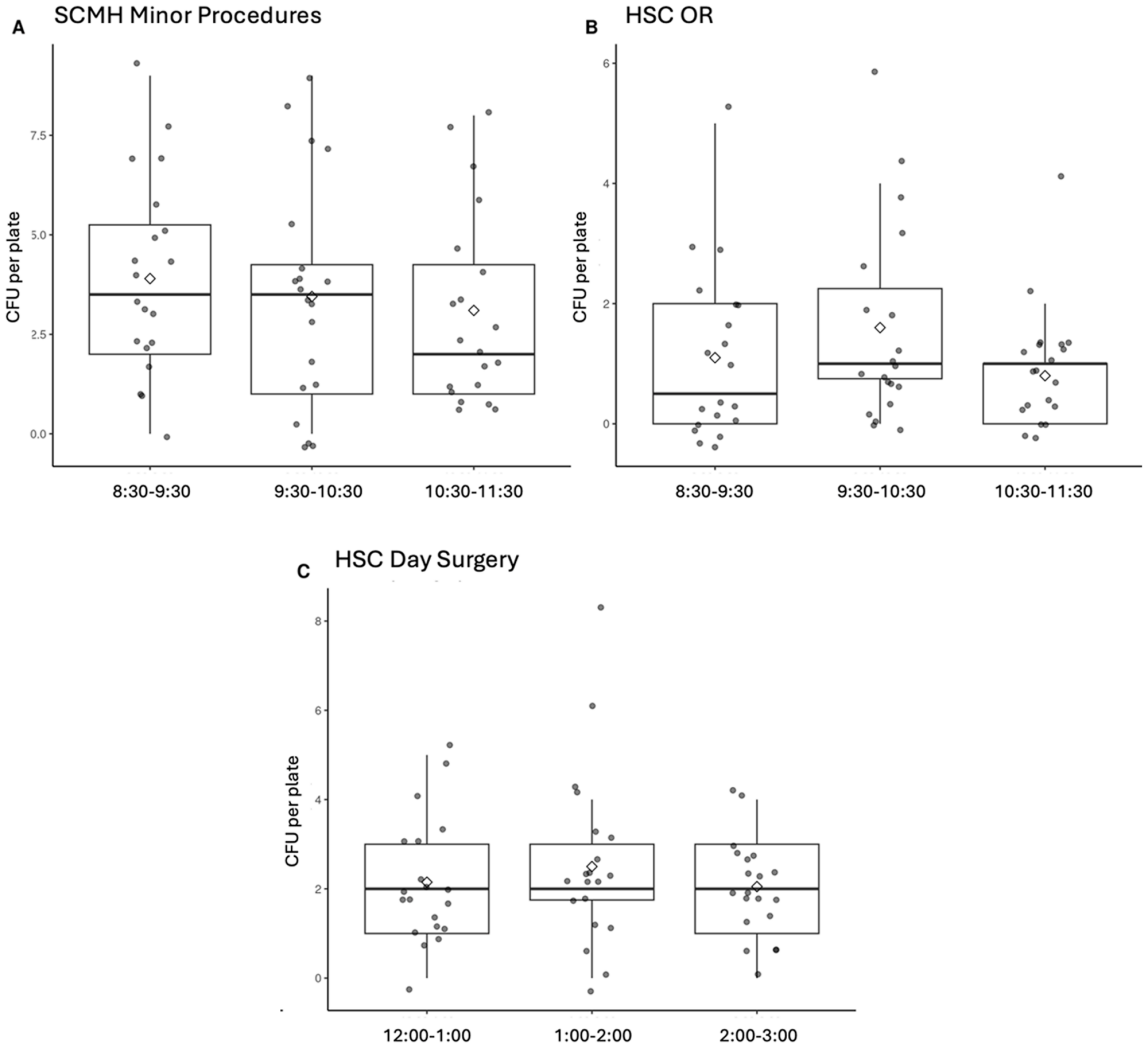
Temporal variation in airborne bacterial contamination (CFU/plate) at St. Clare’s Mercy Hospital (SCMH) minor procedures (A), Health Sciences Centre (HSC) operating room (B), and Health Sciences Centre (HSC) day surgery (C) in Eastern Canada. Box = interquartile range; horizontal line = median; diamond = mean; points = individual settle plates. CFU indicates colony-forming unit; OR, operating room.

Hourly sampling demonstrated consistent patterns within each environment. Higher CFU counts were observed more frequently in the minor procedures room across sampling days, whereas the operating room maintained consistently low counts across time intervals. The day surgery suite demonstrated intermediate contamination levels between the 2 environments. Temporal variation across sampling intervals is illustrated in Figure 2.

Post hoc pairwise comparisons using Tukey HSD demonstrated statistically significant differences between all environments, as detailed in Table 4. The minor procedures room showed higher contamination compared with the operating room (mean difference = 2.32 CFU per plate, adjusted *P* < .0001) and the day surgery suite (mean difference = 1.25 CFU per plate, adjusted *P* = .0013). The day surgery suite also demonstrated higher contamination compared with the operating room (mean difference = 1.07 CFU per plate, adjusted *P* = .0058). These results demonstrate a graded pattern of airborne microbial deposition across environments, with the highest counts in the minor procedures room, intermediate levels in the day surgery suite, and the lowest counts in the operating room.

**Table 4.** Tukey HSD Post Hoc Pairwise Comparisons.

Comparison	Mean difference	Standard error	Tukey *q*	Adjusted *P*-value	Result
Minor procedures vs OR	2.3166	0.2412	9.60	<.0001	Significant
Minor procedures vs day surgery	1.2500	0.2412	5.18	.0013	Significant
Day surgery vs OR	1.0666	0.2412	4.42	.0058	Significant

Abbreviations: HSD, honestly significant difference; OR, operating room.

## Discussion

This study demonstrates a clear gradient of airborne microbial deposition across 3 procedural environments, with the highest CFU counts observed in the minor procedures room and the lowest counts observed in the operating room. These differences occurred despite standardized patient preparation and draping practices across sites, suggesting that environmental conditions within procedural spaces may influence airborne microbial deposition.

Our findings should be interpreted in the context of previous studies demonstrating the safety of field sterility in minor hand procedures. Yu et al^10^ reviewed sterility practices in hand surgery and found no evidence that operating room measures such as gowns, laminar airflow, or full draping reduce SSI rates in minor procedures, concluding that field sterility is appropriate in many settings.^18^ Similarly, Leblanc et al^14^ conducted a multicenter prospective study of 1504 carpal tunnel releases performed in minor procedure rooms and reported a superficial infection rate of 0.4% with no deep infections. Avoricani et al^15^ reported comparable findings in a series of WALANT procedures, with no infections identified at 14 days. These studies support the clinical safety of field sterility in appropriate settings.

Variability in airborne microbial burden across health care environments has been well described. Kauch et al^19^ demonstrated that microbial loads may vary substantially between health care facilities and are influenced by characteristics of the built environment and infection control practices. Previous work has also emphasized that ventilation performance and operating room practices may influence contamination levels within surgical spaces.^12,16,20^ Bischoff et al^13^ reported that laminar airflow systems reduce airborne contamination, although their meta-analysis did not demonstrate consistent reductions in SSI rates across all surgical specialties. These findings suggest that differences in airborne microbial deposition between procedural spaces may reflect differences in environmental infrastructure rather than differences in draping practices alone.

Prior work has also validated the use of passive air sampling for assessing microbial deposition in clinical environments. Napoli et al^21^ demonstrated that both active and passive air sampling correlate with operating theater air quality, although passive sampling may better reflect natural microbial settling without disturbing airflow. Pasquarella et al^17^ further described the IMA, which standardizes passive sampling using the “1/1/1” principle applied in the present study. These reports support the methodological validity of the sampling approach used in this study.

Cost and environmental sustainability are also relevant considerations in surgical sterility practices.^22,23^ Kalliainen and Lacey demonstrated that reducing draping for routine hand procedures can produce meaningful cost savings without increasing infection rates.^23^ Similarly, Yu et al^10^ highlighted that the resource use associated with full operating room sterility may not be necessary for many minor procedures.^11^ Our findings suggest that while reduced sterility protocols may remain appropriate for selected procedures, environmental characteristics of the procedural space should also be considered.

Our findings do not contradict this literature but rather highlight that field-sterile environments are not necessarily uniform. Although both minor procedures and the day surgery suite used the same towel-drape field sterility technique, airborne microbial deposition differed between these 2 environments. This suggests that factors beyond draping technique may influence microbial deposition within procedural spaces.

This study has several limitations. Colony-forming unit counts represent a surrogate measure of airborne microbial deposition rather than a direct measure of surgical site infection risk. The study did not measure airflow characteristics, room traffic, patient masking, or other environmental variables that may influence airborne contamination.^16,22^ Sampling was also performed on consistent weekly days within each environment, and day-to-day variation in surgical activity or personnel could potentially influence microbial counts. These factors limit the ability to attribute observed differences to specific environmental causes.

## Conclusions

This study demonstrates significant differences in airborne microbial deposition across 3 procedural environments, with the lowest contamination observed in the operating room and the highest in the minor procedures room. Notably, microbial deposition differed between the minor procedures room and the day surgery suite despite the use of identical field-sterility draping, suggesting that room-level environmental factors may play an important role in determining airborne microbial burden. These findings indicate that procedural environments employing similar sterility techniques are not necessarily equivalent in terms of airborne contamination. Further investigation into the environmental characteristics influencing airborne microbial deposition in procedural spaces may help guide future infection prevention practices in minor surgical settings.

## Supplemental Material

sj-docx-1-han-10.1177_15589447261467940 – Supplemental material for Airborne Bacterial Deposition Onto Surgical Sites Under Operating Room Versus Field Sterility: A Passive Air Sampling Study Across 3 Surgical Environments Supplemental material, sj-docx-1-han-10.1177_15589447261467940 for Airborne Bacterial Deposition Onto Surgical Sites Under Operating Room Versus Field Sterility: A Passive Air Sampling Study Across 3 Surgical Environments by Erin Hopkins and Joshua Gillis in HAND
