# Supplementary material for: Airborne Bacterial Deposition Onto Surgical Sites Under Operating Room Versus Field Sterility: A Passive Air Sampling Study Across 3 Surgical Environments
Source: Hand (N Y). 2026 Jul 29:15589447261467940. Online ahead of print. doi: 10.1177/15589447261467940 (PMC13420114; doi:10.1177/15589447261467940)
Supplement: sj-docx-1-han-10.1177_15589447261467940 – Supplemental material for Airborne Bacterial Deposition Onto Surgical Sites Under Operating Room Versus Field Sterility: A Passive Air Sampling Study Across 3 Surgical Environments [file sj-docx-1-han-10.1177_15589447261467940.docx]

**Supplemental Material**

**Supplementary Table 1.** Complete dataset of colony-forming unit (CFU) counts from all 180 blood agar plates collected across three hospital environments, organized by date, time interval, and plate number.

| Environment | Date | Time | Plate 1 | Plate 2 | Plate 3 | Plate 4 | Plate 5 |
| --- | --- | --- | --- | --- | --- | --- | --- |
| Minor Procedures | 2025-03-03 | 8:30-9:30 | 8 | 7 | 9 | 2 | 6 |
| Minor Procedures | 2025-03-03 | 9:30-10:30 | 4 | 4 | 4 | 5 | 3 |
| Minor Procedures | 2025-03-03 | 10:30-11:30 | 7 | 6 | 5 | 2 | 3 |
| Minor Procedures | 2025-03-10 | 8:30-9:30 | 1 | 1 | 3 | 5 | 0 |
| Minor Procedures | 2025-03-10 | 9:30-10:30 | 0 | 0 | 2 | 3 | 0 |
| Minor Procedures | 2025-03-10 | 10:30-11:30 | 1 | 3 | 1 | 2 | 3 |
| Minor Procedures | 2025-03-24 | 8:30-9:30 | 4 | 3 | 3 | 4 | 5 |
| Minor Procedures | 2025-03-24 | 9:30-10:30 | 8 | 9 | 7 | 7 | 3 |
| Minor Procedures | 2025-03-24 | 10:30-11:30 | 4 | 8 | 1 | 1 | 8 |
| Minor Procedures | 2025-04-07 | 8:30-9:30 | 2 | 2 | 4 | 2 | 7 |
| Minor Procedures | 2025-04-07 | 9:30-10:30 | 4 | 1 | 1 | 0 | 4 |
| Minor Procedures | 2025-04-07 | 10:30-11:30 | 2 | 1 | 2 | 1 | 1 |
| Operating Room | 2025-03-12 | 8:30-9:30 | 0 | 0 | 0 | 0 | 0 |
| Operating Room | 2025-03-12 | 9:30-10:30 | 0 | 0 | 2 | 1 | 1 |
| Operating Room | 2025-03-12 | 10:30-11:30 | 0 | 1 | 0 | 0 | 0 |
| Operating Room | 2025-03-19 | 8:30-9:30 | 2 | 1 | 3 | 1 | 0 |
| Operating Room | 2025-03-19 | 9:30-10:30 | 3 | 4 | 1 | 3 | 6 |
| Operating Room | 2025-03-19 | 10:30-11:30 | 4 | 1 | 1 | 0 | 1 |
| Operating Room | 2025-03-26 | 8:30-9:30 | 2 | 2 | 5 | 2 | 0 |
| Operating Room | 2025-03-26 | 9:30-10:30 | 4 | 1 | 2 | 0 | 1 |
| Operating Room | 2025-03-26 | 10:30-11:30 | 1 | 0 | 0 | 2 | 1 |
| Operating Room | 2025-04-02 | 8:30-9:30 | 1 | 0 | 0 | 3 | 0 |
| Operating Room | 2025-04-02 | 9:30-10:30 | 1 | 1 | 1 | 0 | 0 |
| Operating Room | 2025-04-02 | 10:30-11:30 | 1 | 1 | 0 | 1 | 1 |
| Day Surgery | 2025-03-06 | 12:00-1:00 | 2 | 1 | 2 | 2 | 3 |
| Day Surgery | 2025-03-06 | 1:00-2:00 | 1 | 0 | 2 | 1 | 3 |
| Day Surgery | 2025-03-06 | 2:00-3:00 | 2 | 1 | 2 | 2 | 3 |
| Day Surgery | 2025-03-20 | 12:00-1:00 | 2 | 1 | 2 | 1 | 0 |
| Day Surgery | 2025-03-20 | 1:00-2:00 | 2 | 2 | 3 | 4 | 2 |
| Day Surgery | 2025-03-20 | 2:00-3:00 | 0 | 1 | 2 | 1 | 2 |
| Day Surgery | 2025-03-27 | 12:00-1:00 | 2 | 1 | 1 | 3 | 1 |
| Day Surgery | 2025-03-27 | 1:00-2:00 | 2 | 2 | 0 | 2 | 1 |
| Day Surgery | 2025-03-27 | 2:00-3:00 | 1 | 4 | 2 | 3 | 1 |
| Day Surgery | 2025-04-03 | 12:00-1:00 | 5 | 4 | 3 | 5 | 2 |
| Day Surgery | 2025-04-03 | 1:00-2:00 | 6 | 2 | 8 | 3 | 4 |
| Day Surgery | 2025-04-03 | 2:00-3:00 | 2 | 3 | 4 | 3 | 2 |
